# Comparing effectiveness of image perturbation and test retest imaging in improving radiomic model reliability

**DOI:** 10.1038/s41598-023-45477-6

**Published:** 2023-10-25

**Authors:** Jiang Zhang, Xinzhi Teng, Xinyu Zhang, Sai-Kit Lam, Zhongshi Lin, Yongyi Liang, Hao Yu, Steven Wai Kwan Siu, Amy Tien Yee Chang, Hua Zhang, Feng-Ming Kong, Ruijie Yang, Jing Cai

**Affiliations:** 1 Department of Health Technology and Informatics, The Hong Kong Polytechnic University, Y920, Lee Shau Kee Building, Hung Hom, Kowloon, Hong Kong, China; 2 Department of Biomedical Engineering, Faculty of Engineering, The Hong Kong Polytechnic University, Hong Kong, China; 3 Research Institute for Smart Ageing, The Hong Kong Polytechnic University, Hong Kong, China; 4 Shenzhen Institute for Drug Control (Shenzhen Testing Center of Medical Devices), Shenzhen, China; 5 Institute of Biomedical and Health Engineering, Chinese Academy of Sciences Shenzhen Institutes of Advanced Technology, Shenzhen, China; 6 Department of Clinical Oncology, The University of Hong Kong, Hong Kong, China; 7 Department of Clinical Oncology, The University of Hong Kong-Shenzhen Hospital, Shenzhen, China; 8 Department of Radiation Oncology, Cancer Center, Peking University Third Hospital, Beijing, China; 9 The Hong Kong Polytechnic University Shenzhen Research Institute, Shenzhen, China; 10 Beijing Linking Medical Technology Co., Ltd., Beijing, China

**Keywords:** Biomarkers, Magnetic resonance imaging

## Abstract

Image perturbation is a promising technique to assess radiomic feature repeatability, but whether it can achieve the same effect as test–retest imaging on model reliability is unknown. This study aimed to compare radiomic model reliability based on repeatable features determined by the two methods using four different classifiers. A 191-patient public breast cancer dataset with 71 test–retest scans was used with pre-determined 117 training and 74 testing samples. We collected apparent diffusion coefficient images and manual tumor segmentations for radiomic feature extraction. Random translations, rotations, and contour randomizations were performed on the training images, and intra-class correlation coefficient (ICC) was used to filter high repeatable features. We evaluated model reliability in both internal generalizability and robustness, which were quantified by training and testing AUC and prediction ICC. Higher testing performance was found at higher feature ICC thresholds, but it dropped significantly at ICC = 0.95 for the test–retest model. Similar optimal reliability can be achieved with testing AUC = 0.7–0.8 and prediction ICC > 0.9 at the ICC threshold of 0.9. It is recommended to include feature repeatability analysis using image perturbation in any radiomic study when test–retest is not feasible, but care should be taken when deciding the optimal feature repeatability criteria.

## Introduction

Radiomics is one of the most up-to-date quantitative imaging techniques nowadays. Quantitative features, which are believed to represent tumor phenotypes that are imperceptible to human eyes, are extracted in a high-throughput manner from routine medical imaging, such as CT, MR, or PET. Morphological, histogram, as well as textural information could be included in different classes of radiomic features. They are then selected and built into different models to help noninvasive diagnosis^1–3^, prognosis^4–6^, and treatment response prediction^7–9^. Despite the promising potential of radiomics, the reliability of radiomic models is one of the major concerns when translating into routine clinical practice.

Radiomic feature repeatability refers to the feature's ability to keep stable when the same subject is imaged several times under the same acquisition settings. It is believed to be the first and foremost criteria to ensure model reliability and has been studied extensively by previous research^10–12^. Test–retest imaging is one of the most popular approaches by repeatedly scanning each patient within a short period of time, and feature repeatability is assessed by comparing the feature values between the two different scans. For example, Granzier et al. identified repeatable radiomic features within breast tissues using a two-day interval test–retest data with fixed scanner and clinical breast protocol^13^. However, test–retest imaging is not a standard clinical procedure and requires additional medical resources and potential extra dose to patients. Consequently, the existing test–retest study include only a limited number of patients, which further reduced the significance of their findings. In addition, the conclusions of feature repeatability are hardly generalizable across image modalities and cancer sites^14^, rendering the necessity of specific repeatability analysis for different radiomic studies.

Several methods have been proposed to assess radiomic feature repeatability through image perturbation. Marco et al. first applied random translations of the regions-of-interest (ROIs) to assess the radiomic feature repeatability on apparent diffusion coefficient (ADC) images^15^. They found an overall satisfactory repeatability and a high site dependency. Zwanenburg et al. proposed to generate pseudo-retest images by random translation, rotation, noise addition and contour randomizations, and demonstrated similar patterns of feature repeatability to test–retest imaging^16^. Further studies have demonstrated the potential of perturbed images in quantifying radiomic model output reliability and improving the model generalizability and robustness by removing low-repeatable features^17–19^. Although perturbation methods have been proven to be capable of capturing the majority of non-repeatable features in test–retest images, it is still unknown if image perturbation could replace test–retest imaging in building a reliable radiomic model.

**Figure 1:**
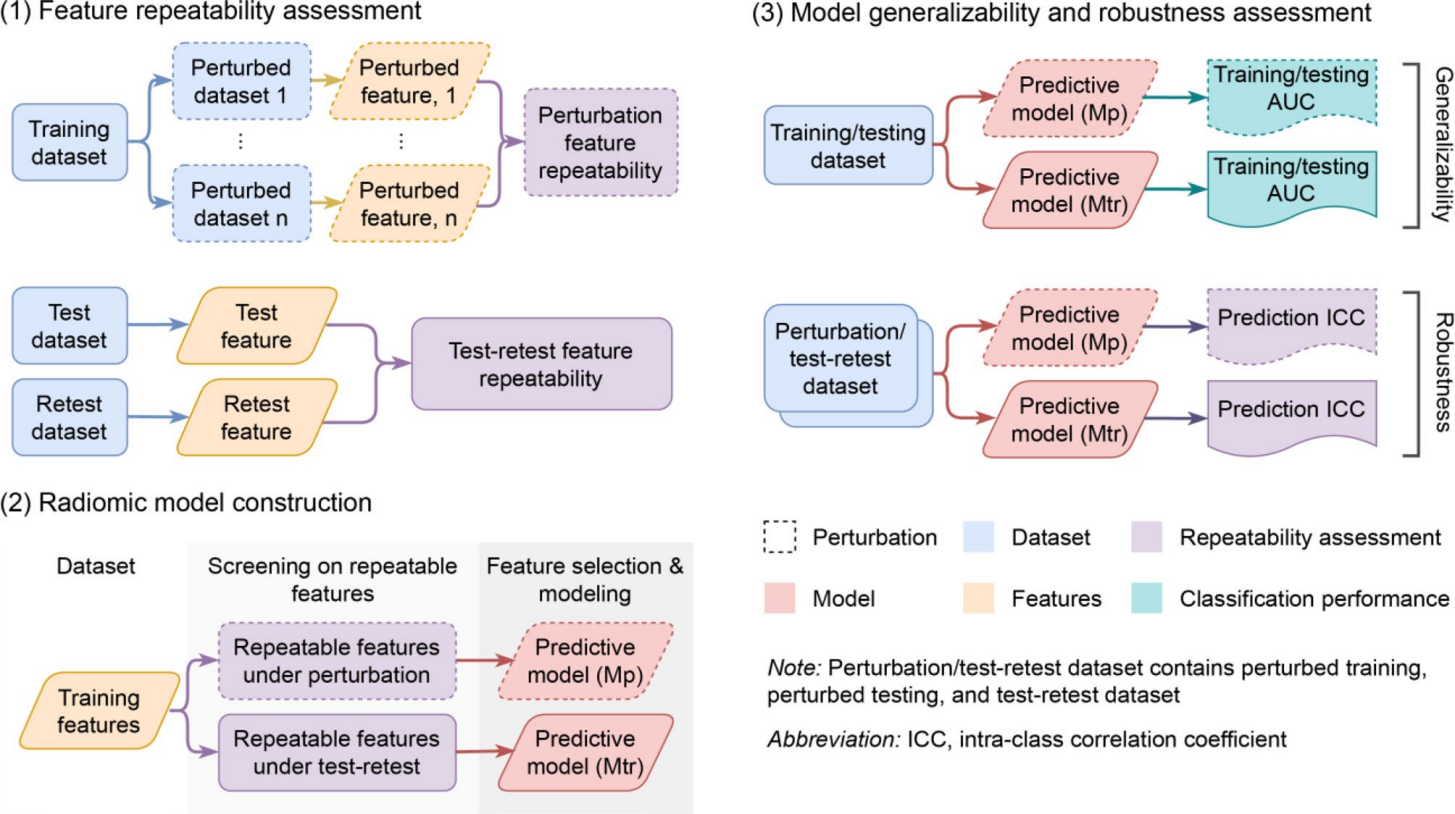
Study workflow. We conducted our study by (1) radiomic feature repeatability assessment by test–retest and image perturbation, (2) radiomic model development using high-repeatable features from the two assessments, and (3) internal generalizability and robustness analysis of the two models. Mp and Mtr are the models based on repeatable features assessed by image perturbation and test–retest respectively.

This study aimed to compare radiomic model reliability after removing non-repeatable radiomic features assessed by image perturbation and test–retest imaging. We retrospectively analyzed a unique breast cancer test–retest ADC image dataset and compared both internal generalizability and robustness of the predictive models on pathological complete response (pCR). The overall study workflow is summarized by Fig. 1. This study could provide the radiomic community direct evidence of the benefit of image perturbation on building reliable radiomic models. Most importantly, whether image perturbation is equivalent to test–retest imaging in building a reliable radiomic model could be directly validated.

## Results

### Feature repeatability and predictability

**Figure 2:**
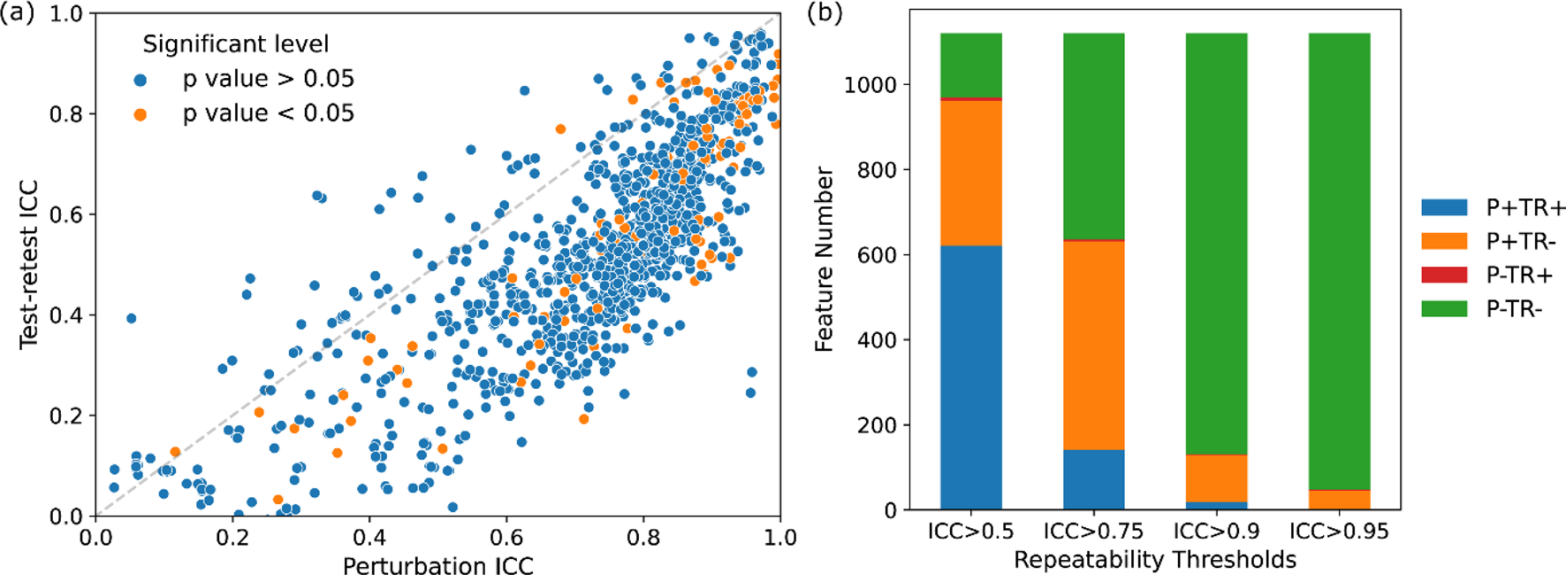
(**a**) Scatter plots showing the repeatability of volume independent features measured by intra-class correlation coefficient (ICC) under test–retest imaging (y-axis) and image perturbation (x-axis). The perturbation method yielded higher ICC values than the test–retest method in general. Furthermore, features that had significant univariate correlations with the outcome, pCR, where colored as orange while the rest as blue. (**b**) Stacked bar plot displaying the feature repeatability agreement between perturbation and test–retest. P + / − indicates the repeatable/unrepeatable feature group by the perturbation method and TR + / − for repeatable/unrepeatable feature group in the test–retest method.

Compared to test–retest, there was a systematic larger feature repeatability on image perturbation. Figure 2a visualizes the distribution of feature ICCs assessed by training perturbation versus test–retest. Among all the 1120 volume-independent radiomic features, only 143 showed lower ICC under image perturbation than test–retest, which can be visualized as scarce scattered points above the diagonal line in Fig. 2a. However, the feature repeatability under image perturbation and test–retest demonstrated a strong correlation with Pearson correlation r = 0.79 (p-value < 0.001).

The feature repeatability agreement between perturbation and test–retest showed a strong dependence on ICC thresholds, as shown in Fig. 2b. In general, the number of commonly repeatable and non-repeatable features between the two ICC measures increased with higher ICC thresholds. Specifically, the number of mutually agreed repeatable features decreased from 621 to 141, 18, and 2 with ICC threshold increased from 0.5 to 0.75, 0.9, and 0.95, as suggested by the shrinking blue bars in Fig. 2b. In contrast, the number of mutually disagreed repeatable features increased from 151 to 484, 989, and 1072 (green bars). For disagreements between perturbation and test–retest evaluation, very few (< 0.7%) features are repeatable against test–retest variations while unrepeatable against perturbation (red bars), and a considerate amount of features are repeatable against perturbation while unrepeatable under test–retest settings (orange bars).

Only a small portion of all the volume-independent radiomic features demonstrated strong univariate correlation with the prediction outcome with an inclination towards high repeatable features (Fig. 2a). Quantitatively, 111 radiomic features reached statistical significance (p-value < 0.05) when correlating with pCR. With the ICC threshold of 0.5, 11% (n = 71) of the high-repeatable features under test–retest had statistical significance and 10% (n = 93). The percentage increased to 23% at ICC threshold of 0.75 but decreased to 5% (n = 1) and 0% (n = 0) at 0.9 and 0.95 for test–retest. However, a continuous increase to 11% (n = 72), 25% (n = 32), and 27% (n = 12) for perturbation was discovered. The final selected features for model development can be found in the Supplementary Material (Table S1).

### Internal generalizability and robustness

**Figure 3:**
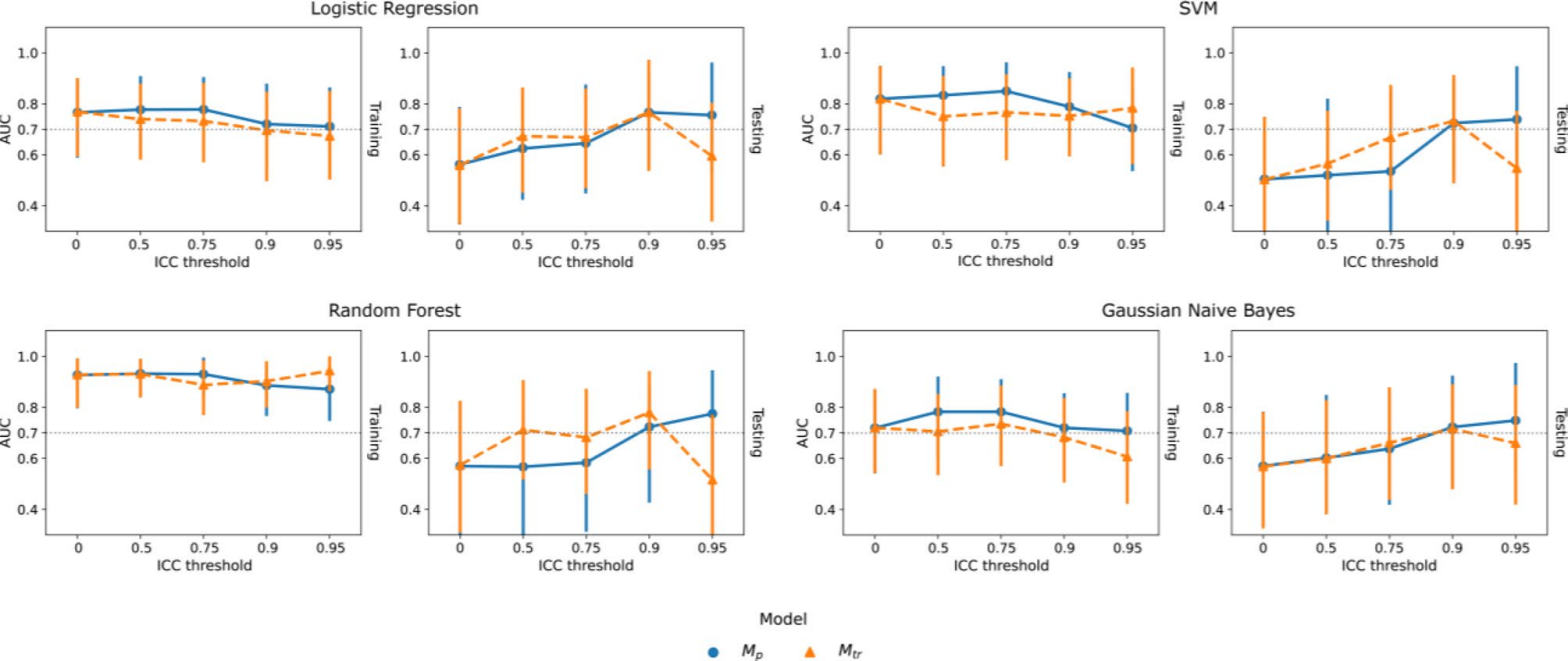
Comparison of internal generalizability between models based on repeatable features assessed by image perturbation (${M}_{p}$, blue) and the test–retest imaging (${M}_{tr}$, orange) under varying thresholds for logistic regression, SVM, random forest, and gaussian naive bayes classifiers. Training and testing classification performance were quantified by area under the receiver operating characteristic curve (AUC). The error bars indicate 95% confidence intervals acquired from 1000-iteration bootstrapping.

**Figure 4:**
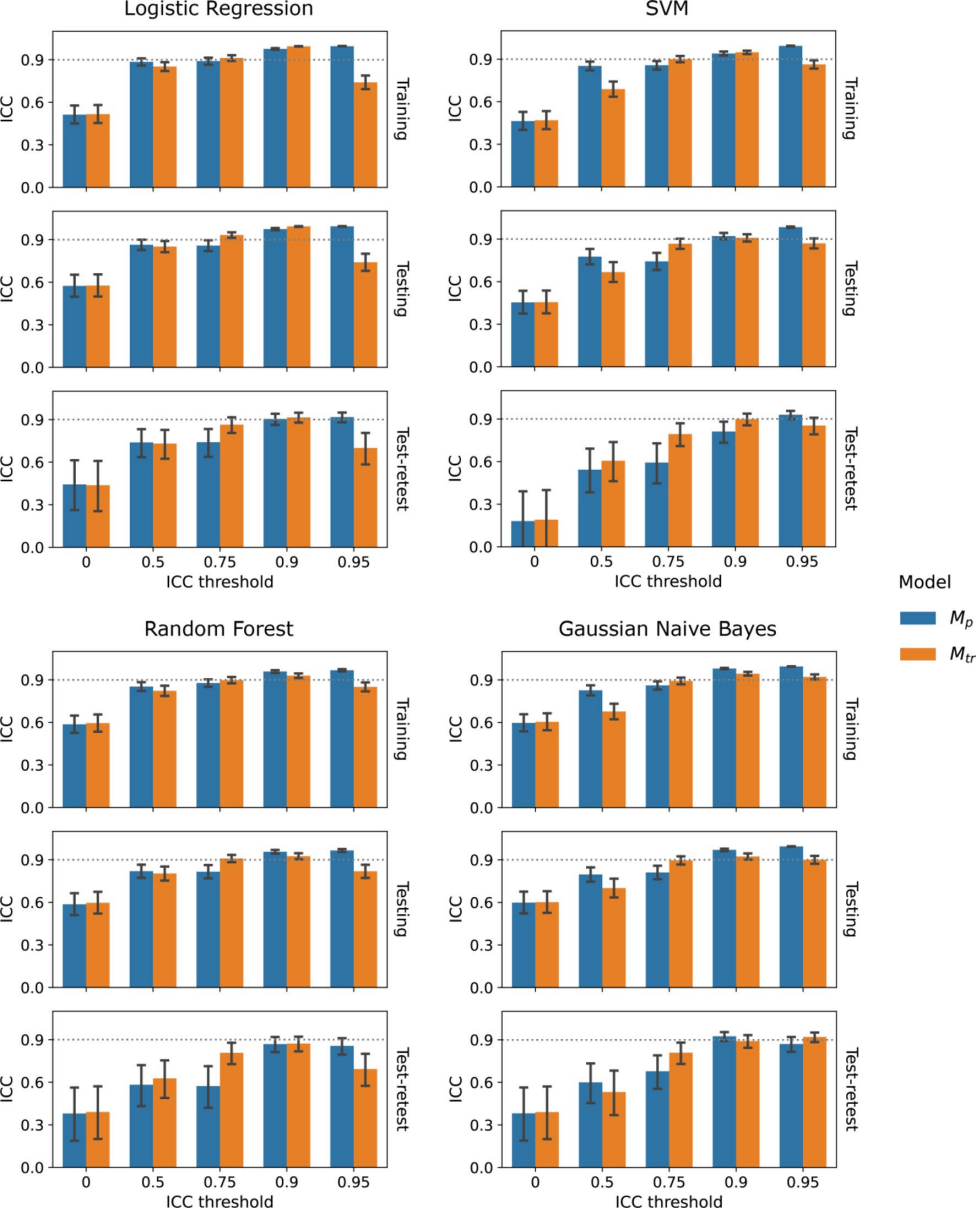
Bar plots for comparing robustness between models based on repeatable features assessed by image perturbation (${M}_{p}$, blue) and the test–retest imaging (${M}_{tr}$, orange) under varying thresholds for logistic regression, SVM, random forest, and gaussian naive bayes classifiers. Model robustness was evaluated by probability prediction ICC under perturbation or tests-retest. The error bars indicate 95% confidence intervals acquired during ICC calculation.

An overall trend of increasing internal generalizability and robustness was observed with increasing ICC thresholds. Figure 3 presented the overall trend and comparisons of training and testing AUCs of ${M}_{p}$ and ${M}_{tr}$ under varying feature ICC thresholds for the four classifiers. For logistic regression, the testing AUC increased significantly from 0.56 (0.41–0.70) at baseline (ICC threshold = 0) to the maximum of 0.76 (0.64–0.88, p = 0.021) at ICC threshold = 0.9 under perturbation and 0.77 (0.64–0.88, p = 0.018) under test–retest. The same trend can be observed for the rest of the classifiers. On the other hand, both ${M}_{p}$ and ${M}_{tr}$ demonstrated steady decreases of the training AUCs under increasing ICC thresholds without statistically significant differences to the baseline. Similarly, the baseline models had the lowest robustness with, for example, prediction ICC = 0.51 (0.45–0.58) on training perturbation, 0.57 (0.49–0.66) on testing perturbation, and 0.45 (0.25–0.62) on test–retest for the logistic regression, as indicated by the lowest bars in Fig. 4. Significant improvement can be already observed when increasing the feature ICC threshold to 0.5 for both ${M}_{p}$ and ${M}_{tr}$.

${M}_{tr}$ demonstrated higher internal generalizability and robustness than ${M}_{p}$ on larger feature ICC filtering thresholds in general. We observed smaller training AUCs and higher testing AUCs of ${M}_{tr}$ at ICC thresholds of 0.5 and 0.75 for all the four classifiers (Fig. 3). The AUC differences between ${M}_{p}$ and ${M}_{tr}$ were kept small with the absolute values below 0.1 (p > 0.05) for logistic regression and gaussian naive bayes while larger differences in testing AUCs were found for SVM and random forest. Under the ICC threshold of 0.75, ${M}_{tr}$ had a significantly higher prediction ICC on both testing perturbation (e.g. logistic regression, ${M}_{tr}$=0.93 (0.91–0.95), ${M}_{p}$=0.86 (0.82–0.90)) and test–retest (${M}_{tr}$=0.87 (0.80–0.92), ${M}_{p}$=0.75 (0.63–0.84)), while smaller differences found on training perturbation (${M}_{tr}$=0.91 (0.89–0.93), ${M}_{p}$=0.90 (0.86–0.92)), as demonstrated by Fig. 3. On the other hand, ${M}_{tr}$ had smaller prediction ICCs under the ICC threshold of 0.5 with significant differences on training and testing perturbation for SVM and gaussian naive bayes. The ICC threshold of 0.9 demonstrated minimum model robustness deviations between ${M}_{p}$ and ${M}_{tr}$, except for SVM under test–retest.

**Figure 5:**
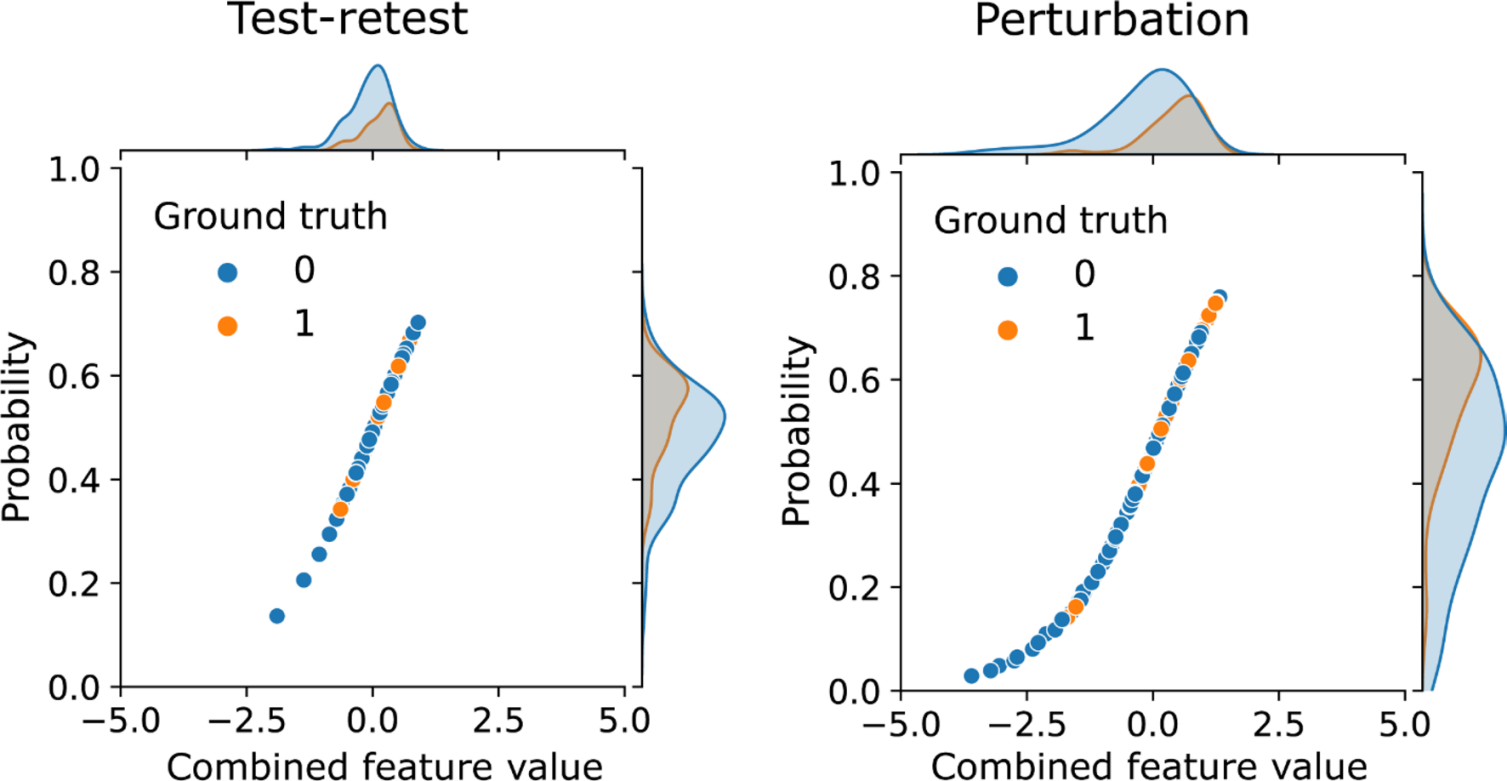
Distributions of the linearly combined feature values and predicted probabilities of the logistic regression models developed from test–retest repeatable features and perturbation repeatable features using the feature intra-class correlation coefficient threshold of 0.95. The predicted probabilities follow the sigmoid mapping of the logistic regression. Samples with ground-truth of non-event are colored by blue and event by orange. Predictions of the test–retest model were aggregated in the high-slop region whereas a wider spread is found for the perturbation model.

Both ${M}_{tr}$ internal generalizability and robustness dropped significantly when increasing the ICC threshold from 0.9 to 0.95 for the four classifiers. For example, for logistic regression, the training AUCs of both ${M}_{p}$ and ${M}_{tr}$ remained stable, while a much larger decrease of testing AUC to 0.59 (0.45–0.73) was found for ${M}_{tr}$ at ICC threshold = 0.95. On the contrary, ${M}_{p}$ had a slightly reduced testing AUC to 0.75 (0.62–0.86). Similar to internal generalizability, the prediction ICC of ${M}_{tr}$ fell significantly on training perturbation, testing perturbation, and test–retest, except for gaussian naive bayes. On the other hand, the prediction ICC of ${M}_{p}$ increased continuously and maximized at ICC threshold = 0.95. Figure 5 presents the distributions of the predicted probabilities of ${M}_{tr}$ and ${M}_{p}$ combining both training and testing samples at ICC threshold of 0.95 using the logistic regression classifier. As expected, they both followed the sigmoid mapping as logistic regression from the linearly combined features values. The predictions of ${M}_{tr}$ are more aggregated in the high-slop region in comparison with ${M}_{p}$ with more spread to the lower tail.

## Discussion

This is the first study that directly compared the reliability of radiomic models based on repeatable radiomic features selected by image perturbation and test–retest imaging using ADC maps derived from a publicly available breast cancer DWI dataset. Model reliability was evaluated in both internal generalizability and robustness, which were quantified by training and testing AUC and probability prediction ICC, respectively. During the experiment, several radiomic models were constructed for comparisons using varying feature repeatability (ICC) thresholds and four different classifiers. We observed systematically lower radiomic feature repeatability assessed by test–retest than perturbation with better binary agreement at higher ICC thresholds. Similar optimal internal generalizability and robustness were achieved by the four classification models based on perturbation (${M}_{p}$) and test–retest (${M}_{tr}$) at the ICC threshold of 0.9 simultaneously. Slightly lower training AUCs and higher testing AUCs were achieved by ${M}_{tr}$ than ${M}_{p}$ at lower ICC thresholds (p > 0.05). In addition, ${M}_{tr}$ demonstrated significantly higher prediction ICCs on training perturbation, testing perturbation, and test–retest at the ICC threshold of 0.75. Notably, increasing the ICC threshold to 0.95 resulted in significant drops of testing AUC and prediction ICCs for ${M}_{tr}$. Our results provide the direct evidence that our perturbation method could replace test–retest method in building a reliable radiomic model with optimal internal generalizability and robustness.

The lower radiomic feature repeatability under test–retest could be largely attributed by the larger variations of tumor segmentations. We further evaluated the segmentation similarities by the Dice similarity coefficients (DSC) and Hausdorff distances (HD) with rigid registrations between test and retest images. The tumor segmentations were less similar between test and retest images (Dice = 0.51(± 0.16), HD = 12.47 mm(± 10.95 mm)) than image perturbation (Dice = 0.71(± 0.11), HD = 2.72 mm(± 0.90 mm)). Previous research by Saha et al. has also suggested less stable radiomics features from breast MRI within the tumor volume due to a large inter-reader variability (Dice = 0.60)^20^. They also emphasized the necessity of standardization in breast tumor segmentation through precise instructions or auto-contouring, where Dice can be increased to 0.77.

The slightly reduced model robustness and internal generalizability on ${M}_{p}$ compared with ${M}_{tr}$ could be explained by the conservative evaluation of feature repeatability by image perturbation. Compared with test–retest, image perturbation yielded larger numbers of repeatable radiomic features using the same ICC filtering thresholds, and more features were found to be predictive in training under univariate test (Fig. 2a). Thus, the final selected features of ${M}_{p}$ were more likely to be predictive in training and resulted in a higher training AUC. On the other hand, ${M}_{p}$ had a slower increase of AUC in testing and prediction ICC in testing perturbation and test–retest before ICC threshold reached 0.95, suggesting a slightly lower internal generalizability and testing robustness than ${M}_{tr}$. The rather strict evaluation of feature repeatability from test–retest may reduce the variabilities of selected features under the same patient condition and enhanced the probability of true discovery. Furthermore, the reduced number of feature candidates could also contribute to the lower risk of overfitting.

However, the extremely high feature ICC threshold of 0.95 resulted in a much lower internal generalizability and robustness under test–retest. During feature selection, only 5 features remained as repeatable for ${M}_{tr}$, and none of them showed significant univariate correlation with pCR in training. Consequently, the final selected features had a minimum probability of being truly predictive, and the constructed model was largely overfitted on training with significantly reduced testing AUC. Meanwhile, the predicted probabilities were confounded within the high-slop region (Fig. 5). Although the selected features and their linear combinations are guaranteed to be highly repeatable (ICC >  = 0.95), they could result in larger variations of the prediction values due to the sigmoid transformation. Although the internal generalizability and robustness describe model reliability from two different perspectives, a model built from features with low sensitivity to the prediction target is more likely to have low performances on both due to the previous discussed reasons. Such findings underline the importance of careful selection on repeatable feature criteria when optimizing the final predictive model. A balance between sensitivity and repeatability needs to be achieved depending on the level of data standardization during application.

Despite the different reliabilities of ${M}_{p}$ and ${M}_{tr}$ under multiple feature repeatability criteria, they both achieved optimal internal generalizability and high robustness at the ICC threshold of 0.9 with similar metric values. Such observation provides the direct evidence that perturbation could replace test–retest imaging while achieving the similar optimal model performance. It is advised to incorporate radiomic feature repeatability analysis using image perturbation when test–retest is less achievable due to limited medical resources. Nevertheless, the optimal ICC threshold discovered by this study may not be generalizable to other radiomics applications where different image modalities and cancer site were studied and different radiomic features were extracted.

In addition to the comparisons between perturbation and test–retest, we discovered a positive impact of higher feature repeatability on model reliability, as suggested by the increasing testing AUCs and prediction ICCs under higher ICC thresholds. Our results are consistent with the findings by Teng et al. that image perturbation could enhance radiomic model reliability on multiple head-and-neck cancer datasets^17^. A higher model output repeatability is generally guaranteed with increased input repeatability when using a linear logistic regression model, as long as the predictability is ensured. Similar to the comparison between ${M}_{p}$ and ${M}_{tr}$, both the reduced feature variabilities and candidate numbers from higher repeatability thresholds could be the major contributors of the enhanced internal generalizability.

Our study has several limitations that need to be addressed by future investigations. First, only one public dataset was used to conduct this experiment. Further investigations on the applicability of our findings need to be conducted on other image modalities, cancer sites, and radiomic feature categories. Second, previous studies have also suggested the impact of scanning settings and image preprocessing parameters on radiomic feature repeatability^21–23^. Therefore, a comprehensive test–retest dataset including different scanners, image acquisition protocols and preprocessing settings is needed to further evaluate the role of perturbation in building a reliable radiomic model. Third, we evaluated model reliability in terms of internal generalizability and robustness without considering external validation performance. Patient data from multiple institutions could be recruited to further enhance our understandings of the impact of feature repeatability on cross-institutional reliability.

## Conclusions

We systematically compared the radiomic model reliability, including both internal generalizability and robustness, between using repeatable radiomic features assessed by image perturbation and test–retest imaging. The same optimal reliability can be achieved by image perturbation as test–retest imaging. Higher feature repeatability resulted in higher model reliability in general, but may have an opposite effect at extremely high repeatability threshold. We recommend the radiomic community to include feature repeatability analysis using image perturbation in any radiomic study when test–retest is not feasible, but care should be taken when deciding the optimal criteria during repeatable feature selection.

## Methods

### Study design

As illustrated in Fig. 1, patients were randomly split into one training and testing set for model development and validation. We assessed model reliability in both internal generalizability and robustness. Internal generalizability was evaluated by its discriminatory power on both the training and testing set. Model robustness was quantified by measuring output probability variability on perturbed training, perturbed testing, and test–retest images, similar to the methodology adopted by Teng et al.^17^. The comparisons were performed under different feature repeatability thresholds to mimic a wide range of selections of repeatability criteria.

### Patient data

We retrospectively collected 191 patients from the publicly available BMMR2 challenge dataset^24,25^. It was derived from the ACRIN 6698 trail where female patients with invasive breast cancer were prospectively enrolled from ten institutions between 2012 and 2015^26^. All the patients received neoadjuvant chemotherapy with 12 weeks of paclitaxel (with/without additional experimental agent) followed by 12 weeks of anthracycline before surgery. Diffusion-weighted MRI was performed for each patient before, 3 weeks after, and 12 weeks after the commencement of the chemotherapy. IRB approval is waived due to the solely use of public data.

Pretreatment DWI-derived ADC maps and manual tumor segmentations were downloaded from The Cancer Imaging Archive^27^ for radiomics model development. Pathologic complete response (pCR), which is the binary outcome assessing the absence of invasive disease in breast and lymph nodes at the time of surgery^28^, was used as the prediction endpoint. We adopted the same train-test split as the BMMR2 challenge with 60% (n = 117) randomly chosen as the training set and the remaining 40% (n = 74) set as the testing set. The same ratios of pCR, hormone receptor (HR), human epidermal growth receptor 2 (HER2) status were controlled for the train-test split. Additionally, we collected the 71 test–retest pre-treatment ADC map pairs scanned within a “coffee-break” from the BMMR2 challenge dataset for comparison. Forty-one test–retest patients overlapped with the primary patient cohort. The tumor volume was manually drawn on dynamic contrast-enhanced MR subtraction images^26^, and migrated to the ADC map.

### Radomics feature extraction

A comprehensive set of Radiomics features was extracted from the original and filtered DWI images within the tumor volume. Before feature extraction, all the DWI images were isotropically resampled to the resolution of 1mmx1mmx1mm and discretized to a fixed bin number of 32 for the original and filtered images. Such preprocessing procedure ensures the consistent image resolution and pixel values across patients with reduced noise. We applied three-dimensional Laplacian-of-Gaussian (LoG) image filters with multiple kernel sizes (1, 2, 3, 4, 5 mm) and eight sets of wavelet filters with full combinations of high-/low-pass in three dimensions. Both first-order (n = 18) and texture features (n = 70) were extracted from each preprocessed image, and shape features (n = 14) were extracted from the tumor segmentation. Texture features include calculations from Gray-Level Co-occurrence Matrix (GLCM), Gray Level Size Zone Matrix (GLSZM), Gray Level Run Length Matrix (GLRLM), and Origi Matrix (NGTDM). The definitions and extraction of radiomic features follow the standardization by the image biomarker standardisation initiative^29^. In total, 1316 radiomics features were extracted for each patient. Detailed settings of the image preprocessing and feature extraction parameters are listed in Table 1. All the image preprocessing and feature extraction procedures were performed by the Python package PyRadiomics (version 3.0.1)^30^.

**Table 1:** Image perturbation, preprocessing, and radiomic feature extraction parameters.

Pixel value offset	0
Resample pixel size (mm)	[1, 1, 1]
Image/mask interpolation algorithm	B-spline
Mask partial volume threshold	0.5
Interpolation grid alignment	Align grid origins
Translation distances (pixel)	[0.0, 0.2, 0.4, 0.6, 0.8]
Rotation angles (degree)	[− 5, 0, 5]
Rotation axis	Mask bounding box center, axial direction
Contour randomization smoothing sigma (mm)	[10, 10, 10]
Contour randomization intensity (mm)	[1, 1, 1]
Perturbation times	40
Image discretization bin number	32
Image filters	Unfiltered, Laplacian-of-Gaussian (3D), Wavelet
Kernel size of Laplacian-of-Gaussian filter (mm)	[1, 2, 3, 4, 5]
Wavelet filter starting level	0
Wavelet filter total level	1
Wavelet filter type	Coilf1
Wavelet filter decompositions	[LLL, HLL, LHL, LLH, LHH, HLH, HHL, HHH]
Feature class	Shape, firstorder, glcm, glrlm, glszm, gldm, ngtdm

### Feature repeatability assessment

Radiomics feature repeatability was assessed from both perturbed images and test–retest images for model reliability comparisons, shown in Fig. 1. We performed 40 image perturbations independently for each patient by random combinations of rotations, translations, and contour randomizations, same as the methodology adopted by Teng et al.^17^. Contour randomization was achieved by deforming the original tumor segmentation by a 3-dimensional random displacement field. The algorithm of random displacement field generation is adapted from the methodology proposed by Simard et al.^31^. A random field vector component on each dimension is generated randomly under a uniform distribution between -1 and 1 for each voxel point. All the z-component of the field vectors on the same slice were kept to the same value to mimic the uniform inter-slice contour variations from the slice-by-slice contouring. The field vectors were then normalized on each dimension by the root mean square and scaled by the user-defined intensity value. They were then smoothed by a gaussian filter with user-defined sigma to ensure the continuous change of the random displacement field and avoid sharp changes of the deformed contours. Detailed image perturbation parameters can be found in Table 1.

The same set of radiomics features were extracted from each perturbed or test/retest image with the same preprocessing procedure. One-way, random, absolute, single rater intraclass correlation coefficient (ICC)^32^ was calculated for each radiomic feature under both image perturbation and test–retest due to the random choice of perturbation parameters and scanning condition for each patient:

$$\frac{M{S}_{R}-M{S}_{W}}{M{S}_{R}+\left(k+1\right)M{S}_{W}},$$

where $M{S}_{R}$ represents the mean square of average perturbation values for patients, $M{S}_{W}$ is the residual source of variance, which is calculated as the variance of perturbation values averaged across patients, and $k$ is the number of perturbations. The ICC calculation was provided by the Python package Pingouin (version 0.5.2)^33^.

### Radiomics model construction

Two types of radiomics models were separately constructed from the repeatable features under image perturbation (${M}_{p}$) and test–retest (${M}_{tr}$), as shown in Fig. 1. Volume dependent radiomic features were first removed to minimize its confounding effect on the comparison results, as tumor volume is more stable by definition. Radiomic features that had a Pearson correlation r > 0.6 to the tumor mesh volume was removed from subsequent analysis. Repeatable features were determined from the pre-set ICC thresholds of 0, 0.5, 0.75, 0.9, and 0.95. They were further filtered by redundancy and outcome relevancy before model training. We adopted the minimum Redundancy—Maximum Relevance (mRMR) feature selection algorithm to rank the repeatable features based on the redundancy and outcome relevancy^34^. Finally, 5 top-ranked features were selected for model development using four different classifiers, including logistic regression, SVM, random forest, and gaussian naive bayes. They were trained by the scikit-learn package (version 1.3.0) in Python. The majority pCR group (non-event) was randomly down-sampled by 500 times and an ensemble of classifiers were trained. The final prediction probability of each patient was given by the average of the individual model predictions. This easy-ensemble approach could reduce the training bias from the heavily imbalanced outcome^35^. It was implemented by the publicly available python package imbalance-learn (version 0.9.1)^36^.

### Model reliability assessment

We assessed radiomics model reliability in both internal generalizability and robustness (Fig. 1). Internal generalizability was assessed by comparing training and testing classification performance evaluated by AUC. Model robustness was assessed by the model prediction repeatability under the setting of both perturbation (training and testing) and test–retest. Probability predictions of either model were generated on the perturbed training, perturbed testing, and test–retest images, and the one-way, random, absolute ICCs were calculated for the prediction repeatability using the same rationale of feature repeatability. Both internal generalizability and robustness were compared between ${M}_{p}$ and ${M}_{tr}$ with different ICC threshold settings, as shown in Fig. 1.

### Statistical analyses

Each classification performance metric was evaluated under 1000-iteration patient bootstrapping to acquire 95% confidence interval (95CI). Two-way p-values for comparing the classification performance were calculated by permutation test with 1000 iterations using the function “permutation_test” provided by the open-source Python package scipy (version 1.9.1)^37^. The comparison was performed between each pair of models with and without feature repeatability filtering as well as ${M}_{p}$ and ${M}_{tr}$. A p-value < 0.05 was considered significant. The 95CI of the model prediction ICC was evaluated according to the formulas presented by McGraw et al. ^32^.

### Supplementary Information

Supplementary Table S1.

## Data Availability

The public dataset of BMMR2 challenge was available via TCIA website (https://wiki.cancerimagingarchive.net/pages/viewpage.action?pageId=50135447).

## Code availability

The extracted radiomics features, analysis results, and code can be accessed from https://github.com/John136219655/BMMR2_test_retest_vs_perturbation.git.
