# Supplementary material for: Comparing effectiveness of image perturbation and test retest imaging in improving radiomic model reliability
Source: Sci Rep. 2023 Oct 25;13:18263. doi: 10.1038/s41598-023-45477-6 (PMC10600245; doi:10.1038/s41598-023-45477-6)
Supplement: Supplementary file 1 — Supplementary Table S1. [file 41598_2023_45477_MOESM1_ESM.pdf]

Table S1. Selected radiomics features for model development

ICC	Perturbation			Test-retest		
	**Image filter**	**Feature class**	**Feature name**	**Image filter**	**Feature class**	**Feature name**
>0	Wavelet (HHH)	ngtdm	Strength	Wavelet (HHH)	ngtdm	Strength
	LoG (sigma=5mm)	firstorder	10Percentile	LoG (sigma=5mm)	firstorder	10Percentile
	Wavelet (LHL)	glcm	JointAverage	Wavelet (LHL)	glcm	JointAverage
	Wavelet (HHH)	firstorder	Skewness	Wavelet (HHH)	firstorder	Skewness
	LoG (sigma=1mm)	glcm	MCC	LoG (sigma=1mm)	glcm	MCC
>0.5	Wavelet (HHH)	ngtdm	Strength	Wavelet (HHH)	ngtdm	Strength
	LoG (sigma=5mm)	firstorder	10Percentile	LoG (sigma=5mm)	firstorder	10Percentile
	Original	shape	Elongation	Original	shape	Skewness
	LoG (sigma=2mm)	glszm	LowGrayLevelZone Emphasis	Original	shape	Elongation
	LoG (sigma=3mm)	firstorder	InterquartileRange	Wavelet (LLH)	glcm	ClusterShade
>0.75	Wavelet (HHH)	ngtdm	Strength	Original	shape	MajorAxisLength
	LoG (sigma=5mm)	firstorder	10Percentile	LoG (sigma=5mm)	firstorder	Median
	Original	shape	Elongation	Wavelet (LLH)	glcm	ClusterShade
	LoG (sigma=3mm)	firstorder	InterquartileRange	LoG (sigma=4mm)	firstorder	Minimum
	Original	glszm	ZoneEntropy	Wavele (HHH)	ngtdm	Coarseness
>0.9	Original	shape	MajorAxisLength	Original	shape	Maximum3DDiameter
	Wavelet (LHH)	firstorder	InterquartileRange	LoG (sigma=5mm)	firstorder	Mean
	LoG (sigma=5mm)	firstorder	Range	Original	firstorder	90Percentile
	Original	shape	Elongation	LoG (sigma=4mm)	gldm	SmallDependenceHigh GrayLevelEmphasis
	Original	shape	Maximum2DDiam eterColumn	Wavelet (LLL)	firstorder	90Percentile
>0.95	Original	shape	MajorAxisLength	Original	firstorder	90Percentile
	LoG (sigma=5mm)	firstorder	Median	LoG (sigma=3mm)	firstorder	Mean
	Original	shape	Maximum2DDiam eterColumn	Wavelet (LLL)	firstorder	90Percentile
	Original	shape	Maximum3DDiam eterColumn	Wavele (LLL)	firstorder	Median
	Original	shape	Maximum2DDiam eterRow	Original	firstorder	Median
